# Long-term Outcomes After Kidney Transplantation From DBD Donors Aged 70 y and Older

**DOI:** 10.1097/TXD.0000000000001660

**Published:** 2024-06-20

**Authors:** Jørn Petter Lindahl, Anders Åsberg, Kristian Heldal, Trond Jenssen, Christina Dörje, Morten Skauby, Karsten Midtvedt

**Affiliations:** 1 Department of Transplant Medicine, Section of Nephrology, Oslo University Hospital, Rikshospitalet, Oslo, Norway.; 2 Department of Pharmacy, University of Oslo, Oslo, Norway.; 3 Institute of Health and Society, University of Oslo, Oslo, Norway.; 4 Institute of Clinical Medicine, University of Oslo, Oslo, Norway.; 5 Department of Transplant Medicine, Section of Transplant Surgery, Oslo University Hospital, Rikshospitalet, Oslo, Norway.

## Abstract

**Background.:**

Transplantation of kidneys from elderly donations after brain death (DBD) donors has increased owing to organ shortages. We aimed to assess the impact on long-term kidney transplant outcomes from DBD donors aged 70 y and older compared with kidneys from younger donors.

**Methods.:**

From 2007 to 2022, 2274 first single kidney transplantations from DBD donors were performed at our center. Data from 1417 kidney transplant recipients receiving a DBD organ were included and categorized into 3 groups according to donor age: 70 y and older (n = 444, median age 74 y), 60–69 y (n = 527, median age 64 y), and a reference group consisting of donors aged 45–54 y (n = 446, median age 50 y). Kaplan-Meier plots and multivariate Cox regression with correction for recipient, donor, and transplant characteristics were used to investigate patient and kidney graft survival outcomes.

**Results.:**

The median patient follow-up time was 9.3 y (interquartile range, 5.3–13.1). The adjusted hazard ratios for patient death in recipients of kidneys from DBD donors aged 70 y and older compared with 60–69 y and 45–54 y were 1.12 (95% confidence interval [CI], 0.92-1.36; *P = *0.26) and 1.62 (95% CI, 1.26-2.07; *P < *0.001), respectively. Compared with recipients of donors aged 60–69 y and 45–54 y, the adjusted hazard ratios for kidney graft loss in recipients of donors aged 70 y and older were 1.23 (95% CI, 1.02-1.48; *P = *0.029) and 1.94 (95% CI, 1.54-2.45; *P < *0.001), respectively.

**Conclusions.:**

Transplantation of kidneys from DBD donors aged 70 y and older resulted in acceptable long-term outcomes and is encouraging.

There is a rising population of older adults living significantly longer than their predecessors. Chronic kidney disease is prevalent in the elderly, leading to an increased number of patients requiring renal replacement therapy, including kidney transplantation.^1^ In Norway, patients aged 70 y and older waiting for a kidney transplant constitute almost 25% of the waitlisted patients.^2^ Kidney transplantation remains the best therapeutic option for most patients with end-stage renal disease, and regardless of age, kidney transplant patients live longer than chronic dialysis patients.^3-7^ Worldwide, there is a discrepancy between the increasing number of patients on waiting lists and the number of transplants performed. Because of the increasing number of elderly people in the general population, older donors will form an increasingly larger proportion of possible deceased kidney donors. The increased utilization of organs from older deceased donors could be an important way to expand the pool of kidney donors, thereby reducing the waiting list. However, increased donor age is, up till now, associated with worse graft survival.^8,9^ Therefore, the extent to which older deceased kidney donors should be used and the maximum age of kidney donors remain questionable.

We investigated the long-term patient and graft survival outcomes in recipients of a single primary kidney obtained from donation after brain death (DBD) donors aged 70 y and older compared with the outcomes of kidney transplant recipients of DBD donors aged 60–69 y and a reference group consisting of kidney transplant recipients of DBD donors aged 45–54 y, that is, donor kidneys most transplant centers accept. We addressed the question of whether the use of 70 y and older DBD kidneys is acceptable with regard to patient and kidney graft survival compared with younger DBD kidneys, thereby an approach to reduce the waiting list.

## MATERIALS AND METHODS

All organ transplantations in Norway are performed at a single center, Oslo University Hospital, Rikshospitalet, in Oslo, which serves a population of approximately 5.5 million inhabitants. Currently, 250–275 kidney transplantations, including DBD, controlled donation after circulatory death (cDCD), and living donation, are performed annually at our center.^2^

### Study Design and Patients

This was a national retrospective single-center study. The data of all patients aged 18 y and older who underwent their first single deceased donor kidney transplantation from DBD between 2007 and 2022 were retrieved from the Norwegian Renal Registry. Patients were selected and grouped into 3 categories based on the kidney donor age: 70 y and older, 60–69 y; and a reference group consisting of donors aged 45–54 y to ensure an equal patient sample size as in the older donor groups and a representation of donor kidneys (median age 50 y) most transplant centers worldwide accept to use. The cases were closed for follow-up analysis on November 29, 2023. Kidney graft loss was defined as a return to long-term dialysis, retransplantation, or patient death with a functioning kidney graft.

### Decision to Accept and Transplant a DBD Kidney

Twenty-eight donor hospitals are distributed across the country. The initial workup of a potential DBD is performed locally. Transplant surgeons make the decision to accept on call. Generally, a potential DBD donor should be cardiovascularly stable and have a creatinine level in the normal range or slightly elevated. Furthermore, urine microscopy should be normal, and urine production should be retained during organ retrieval. In donors with risk factors and elevated creatinine levels, the estimated glomerular filtration rate (eGFR) is calculated using the Crockcroft and Gault formula, and if eGFR is <60 mL/min, the kidneys are not automatically accepted for transplantation. We always ask family relatives or the next of kin to the deceased whether they accept organ donation. This is done before deciding to proceed and perform a workup for DBD/cDCD. If 1 family member says no, the donation is not fulfilled. This occurs in approximately 25% of potential deceased donors. We do not perform renal biopsies to decide whether to use a potential DBD kidney. This has previously been addressed by our group, focusing on organs from DBD donors older than 75 y.^10^

Once a DBD kidney is offered to the national transplant center, it is rarely discarded, defined as procured but not transplanted. In 2023, there were 125 actual deceased donors (104 DBD/21 cDCD). One hundred one DBD donors were used (1 discarded because of cancer, 1 technical failure during procurement, and 1 technical failure for other reasons). In a potential DBD donor aged 70 y and older, comorbidities such as hypertension and diabetes in the donor will more often end up as not being “put forward/offered,” more often in female than in male donors. A potential DBD donor younger than 70 y is almost always accepted, even with comorbidities, such as hypertension and diabetes, as long as the eGFR is >60 mL/min.

### Immunosuppressive Therapy

The majority of recipients received basiliximab as induction therapy in addition to a calcineurin inhibitor (CNI), tacrolimus, and sometimes cyclosporine, in combination with an antimetabolite, mycophenolate mofetil, and a glucocorticoid, prednisolone. CNIs were administered according to the ELITE-Symphony study, which aimed for low CNI trough values.^11^

### Statistical Analysis

Demographic data were summarized and categorized according to donor age groups. Continuous variables were reported as median (interquartile range) because of nonnormal distribution. Categorical data were described using frequencies. The independent samples Kruskal-Wallis test was used to compare continuous variables. Categorical variables were compared using the Pearson χ^2^ test (Fisher exact test was applied if the number of observations per cell was <5). Kaplan-Meier curves were used to construct probability estimates of patient and kidney graft survival. Cox proportional hazards models were used to determine the unadjusted and adjusted HRs for patient death and kidney graft loss. Patients who received kidney transplants from DBD deceased donors aged 45–54 y were used as references. The association between donor age and patient death and kidney graft loss was assessed after adjusting for the recipient, donor, and transplant risk factors. The proportional hazard assumption was tested through graphical checks. All significant risk factors (*P* < 0.1) in the univariate analysis were included in the multivariate analysis. All reported *P* values were 2-tailed; *P* values of <0.05 were considered significant. Statistical analyses were conducted using IBM SPSS Statistics for Windows, version 29 (IBM, Armonk, NY) or Stata version 18 (StataCorp LP, College Station, TX).

### Missing Data

No patient was lost to follow-up. Donor body mass index (BMI), last donor creatinine level, and cold ischemia time (CIT) were the only variables with some missing data. Of the 1417 transplantations, missing data were observed in 16 (<1%) for donor BMI, 2 (<1%) for the last donor creatinine level, and 2 (<1%) for CIT data. Because there were few missing data points, missing data were replaced by the mean of all observed values for the same donor age group variable instead of using multiple imputation methods for complete data analysis.

## RESULTS

**FIGURE 1.**
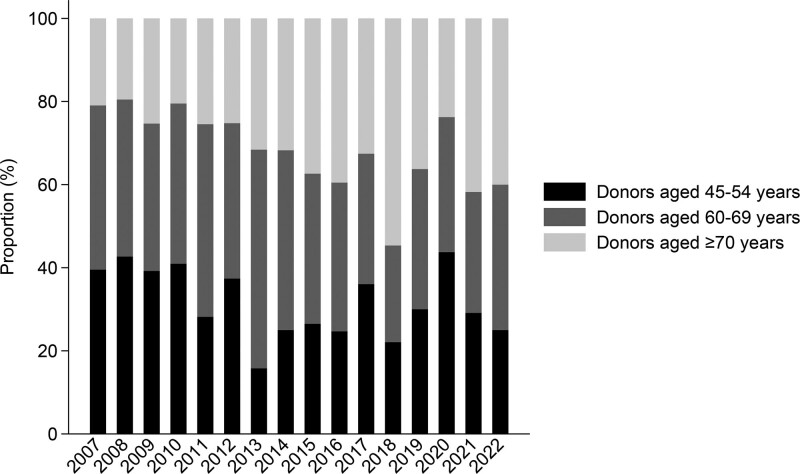
The proportions (%) of DDs aged 70 y and older increased from 2007 to 2022 in the investigated donor groups. DD, deceased donor.

From 2007 to 2022, a total of 2274 DBD first single kidney transplants were performed at our center. In total, data from 1417 kidney transplant recipients receiving a DBD organ were included and categorized into 3 groups according to donor age at the time of death: 70 y and older (n = 444, median age 74 y), 60–69 y (n = 527, median age 64 y), and 45–54 y (n = 446, median age 50 y). The demographic features of the study population are shown in Table 1. The kidney transplant recipients not included received a DBD from donors younger than 45 y (n = 616) or 55–59 y (n = 241). The proportion of deceased kidney donors aged 70 y and older has increased significantly over time. In 2007, 21% of all deceased kidney donors were aged 70 y and older compared with 40% in 2022 in the investigated donor groups (Figure 1). As expected, recipients of donor kidneys aged 70 y and older were older (median age 71 y) than recipients of donor kidneys aged 60–69 y (median age 63 y) and recipients of donor kidneys aged 45–54 y (median age 56 y).

**TABLE 1.** Recipient, donor, and transplant characteristics at transplantation by the donor age group

Variable	Donor age group			*P*
	45–54 y (N = 446)	60–69 y (N = 527)	≥70 y (N = 444)	
Recipient characteristics				
Age, y	56 (48–62)	63 (57–70)	71 (65–74)	<0.001
Male sex, n (%)	295 (66)	348 (66)	292 (66)	0.99
Pretransplant history of, n (%)				
Diabetes	260 (58)	290 (55)	278 (63)	0.058
Heart disease	145 (33)	204 (39)	193 (44)	0.003
Vascular disease	34 (8)	64 (12)	56 (13)	0.028
Treated cancer	35 (8)	69 (13)	93 (21)	<0.001
CMV seropositive	330 (74)	400 (76)	349 (79)	0.27
Pretransplant				
Time on dialysis, mo	22.0 (13.3–35.4)	24.5 (14.7–39.1)	23.1 (12.9–34.5)	0.16
Time on waiting list, mo	13.8 (7.3–22.2)	13.6 (7.1–22.3)	15.0 (7.8–25.8)	0.10
Mode of first RRT, n (%)				
Transplantation	90 (20)	98 (19)	96 (22)	0.50
Hemodialysis	260 (58)	324 (61)	253 (57)	0.34
Peritoneal dialysis	96 (22)	105 (20)	95 (21)	0.79
Donor characteristics				
Age, y	50 (47–53)	64 (62–67)	74 (72–77)	
Male sex, n (%)	254 (57)	332 (63)	214 (48)	<0.001
BMI, kg/m^2^	25.6 (23.4–27.8)	25.7 (23.4–28.3)	25.1 (23.1–27.8)	0.13
Last creatinine, mg/dL	0.77 (0.61–1.00)	0.76 (0.59–0.99)	0.74 (0.59–0.90)	0.077
Cause of death, n (%)				
Cerebrovascular	264 (59)	390 (74)	393 (89)	<0.001
Other	182 (41)	137 (26)	51 (12)	<0.001
Transplant characteristics, n (%)				
≥1 HLA-DR mismatches	311 (70)	349 (66)	318 (72)	0.18
HLA class I and/or II PRA^*a*^	86 (19)	100 (19)	69 (16)	0.26
Cold ischemia time, h	13.7 (10.8–17.2)	13.4 (10.1–17.2)	12.0 (9.1–15.4)	<0.001

Data are presented as median (IQR) or frequencies (%).

^*a*^HLA class I and/or II PRA describe the number (%) of patients with PRA ≥20%.

BMI, body mass index; CMV, cytomegalovirus; IQR, interquartile range; PRA, panel-reactive antibody; RRT, renal replacement therapy.

### Patient Survival

**FIGURE 2.**
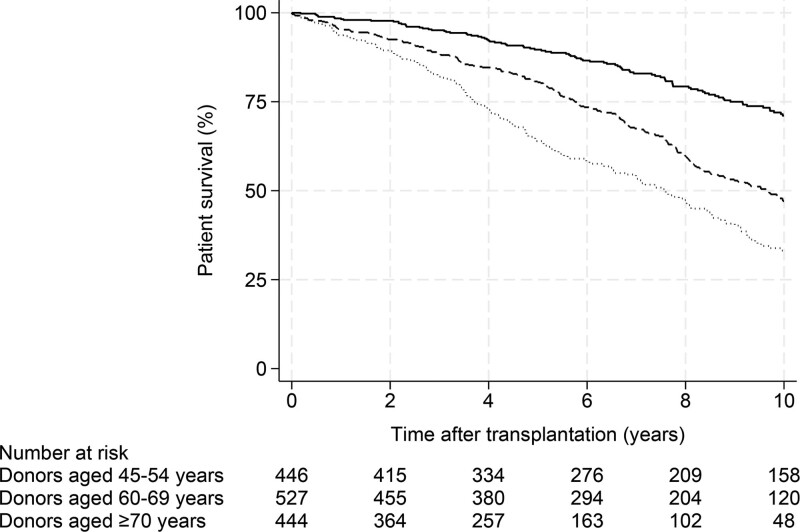
Kaplan-Meier probability estimates of patient survival, grouped by donor age (*P < *0.001). Donors aged 45–54 y, solid line; 60–69 y, dashed line; and 70 y and older, dotted line.

The median patient follow-up time after transplantation was 9.3 y (interquartile range, 5.3–13.1). Patient survival rates at 1, 3, 5, and 10 y posttransplantation according to the donor age group are presented in Table 2. During the study period, a total of 592 patients died of all causes (recipients of donors aged 70 y and older, n* = *229 [52%]; recipients of donors aged 60–69 y, n* = *243 [46%]; and recipients of donors aged 45–54 y, n* = *120 [27%]). Kaplan-Meier curves for patient death by donor age group are shown in Figure 2. The median patient survival after transplantation was 7.6 y for recipients of donors aged 70 y and older. The median patient survival time was not reached for recipients of donors aged 60–69 and 45–54 y.

**TABLE 2.** Estimated survival probability (95% CI) at 1, 3, 5, and 10 y by the donor age group

Variable	Donor age group		
	45–54 y (N = 446)	60–69 y (N = 527)	≥70 y (N = 444)
Patient survival at			
1 y	98 (97-99)	95 (93-97)	94 (91-96)
3 y	95 (93-97)	89 (85-91)	82 (78-86)
5 y	90 (86-92)	80 (77-84)	64 (59-69)
10 y	71 (66-76)	47 (42-52)	33 (27-39)
Graft survival at			
1 y	97 (95-98)	93 (90-95)	89 (85-91)
3 y	93 (90-95)	86 (83-89)	78 (73-81)
5 y	87 (83-90)	76 (72-79)	59 (54-64)
10 y	65 (59-70)	40 (35-45)	29 (23-35)
Death-censored graft survival at			
1 y	99 (97-100)	97 (96-98)	93 (91-95)
3 y	97 (95-98)	96 (94-97)	92 (88-94)
5 y	95 (93-97)	93 (90-95)	87 (83-90)
10 y	89 (84-92)	82 (77-86)	80 (73-86)

CI, confidence interval.

When comparing recipients of donors aged 70 y and older and 60–69 y with the reference group consisting of recipients of donors aged 45–54 y, the hazard ratios (HRs) from unadjusted Cox models for all-cause mortality were 3.42 (95% confidence interval [CI], 2.73-4.29; *P < *0.001) and 2.12 (95% CI, 1.70-2.64; *P < *0.001), respectively (Table 3). The unadjusted HR and 95% CI for all-cause mortality in recipients of donors aged 70 y and older compared with recipients who received kidneys from donors aged 60–69 y were 1.61 (95% CI, 1.34-1.93; *P < *0.001).

**TABLE 3.** HRs for patient death by donor age group

Variable	Unadjusted			Adjusted		
	HR	95% CI	*P*	HR	95% CI	*P*
Recipient age (per year)	1.08	1.07-1.09	<0.001	1.07	1.06-1.09	<0.001
Male recipient	1.25	1.05-1.50	0.013	1.16	0.97-1.39	0.11
Diabetes	1.03	0.87-1.22	0.72			
CMV seropositive recipient	1.30	1.06-1.59	0.010	0.99	0.81-1.21	0.90
Time on dialysis (per year)	1.12	1.08-1.17	<0.001	1.16	1.11-1.21	<0.001
Donor age group						
45–54 y		Reference			Reference	
60–69 y	2.12	1.70-2.64	<0.001	1.50	1.20-1.88	<0.001
≥70 y	3.42	2.73-4.29	<0.001	1.62	1.26-2.07	<0.001
Male donor	0.93	0.79-1.10	0.39			
≥1 HLA-DR mismatches	1.07	0.90-1.26	0.46			
HLA class I and/or II PRA	0.93	0.74-1.15	0.49			
Cold ischemia time (per hour)	0.99	0.98-1.01	0.44			
Delayed graft function	1.36	1.07-1.73	0.013	1.32	1.03-1.68	0.026

CI, confidence interval; CMV, cytomegalovirus; HR, hazard ratio; PRA, panel-reactive antibody.

Multivariate Cox proportional hazards models were adjusted for recipient, donor, and transplant risk factors as outlined in Table 3. Recipient age, dialysis duration, donor age, and delayed graft function (DGF) were inversely associated with long-term patient survival. The adjusted HRs and 95% CIs for all-cause mortality in kidney recipients of donors aged 70 y and older were 1.62 (95% CI, 1.26-2.07; *P < *0.001) and 1.50 (95% CI, 1.20-1.88; *P < *0.001) in recipients who received kidneys from donors aged 60–69 y, respectively, using kidney recipients aged 45–54 y, with donors as reference. The adjusted HR and 95% CI for all-cause mortality in kidney recipients of donors aged 70 y and older compared with recipients who received kidneys from donors aged 60–69 y were 1.12 (95% CI, 0.92-1.36; *P = *0.26).

### Kidney Graft Survival

**FIGURE 3.**
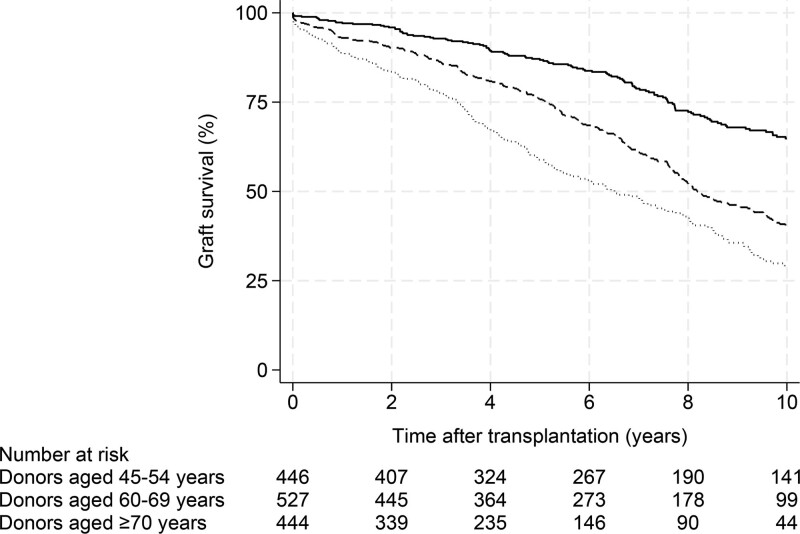
Kaplan-Meier probability estimates of kidney graft survival, grouped by donor age (*P < *0.001). Donors aged 45–54 y, solid line; 60–69 y, dashed line; and 70 y and older, dotted line.

Kidney graft survival at 1, 3, 5, and 10 y posttransplantation according to donor age group is shown in Table 2. In total, 669 death-uncensored kidney graft losses were recorded (recipients of donors aged 70 y and older, n* = *250 [56%]; recipients of donors aged 60–69 y, n* = *276 [52%]; and recipients of donors aged 45–54 y, n* = *143 [32%]). There were no differences between groups for DGF (*P = *0.98), which was observed in 169 kidney recipients (70 y and older, n* = *52 [12%], 60–69 y, n* = *64 [12%], and 45–54 y, n* = *53 [12%]). Kaplan-Meier curves for kidney graft survival according to donor age group are shown in Figure 3. Median kidney graft survival was 6.5 y in kidney recipients of donors aged 70 y and older and 8.2 y in kidney recipients of donors aged 60–69 y. The median kidney graft survival was not achieved in kidney recipients of donors aged 45–54 y.

When comparing kidney recipients of donors aged 70 y and older and 60–69 y with the reference group consisting of kidney recipients of donors aged 45–54 y, the HRs from unadjusted Cox models for kidney graft loss were 3.15 (95% CI, 2.55-3.88; *P < *0.001) and 2.06 (95% CI, 1.68-2.52; *P < *0.001), respectively (Table 4). The unadjusted HR and 95% CI for kidney graft loss in kidney recipients of donors aged 70 y and older compared with recipients who received kidneys from donors aged 60–69 y were 1.52 (95% CI, 1.28-1.81; *P < *0.001).

**TABLE 4.** HRs for kidney graft loss by donor age group

Variable	Unadjusted			Adjusted		
	HR	95% CI	*P*	HR	95% CI	*P*
Recipient age (per year)	1.06	1.05-1.06	<0.001	1.04	1.04-1.05	<0.001
Male recipient	1.22	1.03-1.44	0.019	1.16	0.98-1.37	0.079
Diabetes	1.23	1.05-1.45	0.010	1.19	1.01-1.40	0.035
CMV seropositive recipient	1.17	0.97-1.41	0.097	0.96	0.80-1.16	0.66
Time on dialysis (per year)	1.12	1.07-1.16	<0.001	1.14	1.09-1.19	<0.001
Donor age group						
45–54 y		Reference			Reference	
60–69 y	2.06	1.68-2.52	<0.001	1.63	1.32-2.01	<0.001
≥70 y	3.15	2.55-3.88	<0.001	1.94	1.54-2.45	<0.001
Male donor	0.97	0.83-1.14	0.74			
≥1 HLA-DR mismatches	1.09	0.93-1.28	0.29			
HLA class I and/or II PRA	1.05	0.86-1.28	0.66			
Cold ischemia time (per hour)	1.00	0.98-1.01	0.59			
Delayed graft function	1.55	1.25-1.94	<0.001	1.39	1.11-1.74	0.004

CI, confidence interval; CMV, cytomegalovirus; HR, hazard ratio; PRA, panel-reactive antibody.

Multivariate Cox proportional hazards models were adjusted for recipient, donor, and transplant risk factors (Table 4). Recipient age, diabetes, dialysis duration, donor age, and DGF were inversely associated with long-term kidney graft survival. The adjusted HRs and 95% CIs for kidney graft loss in kidney recipients of donors aged 70 y and older were 1.94 (95% CI, 1.54-2.45; *P < *0.001) and 1.63 (95% CI, 1.32-2.01; *P < *0.001) in recipients who received kidneys from donors aged 60–69 y, respectively, using kidney recipients of donors aged 45–54 y as reference. The adjusted HR and 95% CI for kidney graft loss in kidney recipients of donors aged 70 y and older compared with recipients who received kidneys from donors aged 60–69 y were 1.23 (95% CI, 1.02-1.48; *P = *0.029).

### Death-censored Kidney Graft Survival

**FIGURE 4.**
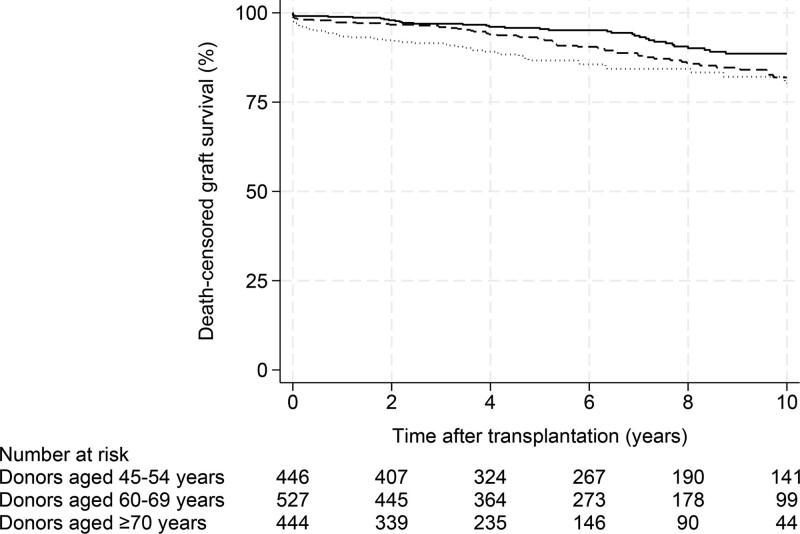
Kaplan-Meier probability estimates of death-censored kidney graft survival, grouped by donor age (*P < *0.001). Donors aged 45–54 y, solid line; 60–69 y, dashed line; and 70 y and older, dotted line.

The death-censored kidney graft survival rates at 1, 3, 5, and 10 y posttransplantation according to the donor age group are shown in Table 2. There were 167 death-censored kidney graft losses (recipients of donors aged 70 y and older, n* = *59 [13%]; recipients of donors aged 60–69 y, n* = *65 [12%]; and recipients of donors aged 45–54 y, n* = *43 [10%]). Kaplan-Meier curves for death-censored kidney graft survival according to donor age group are shown in Figure 4.

When comparing kidney recipients of donors aged 70 y and older and 60–69 y with the reference group, the HRs from unadjusted Cox models for death-censored kidney graft loss were 2.25 (95% CI, 1.51-3.36; *P < *0.001) and 1.56 (95% CI, 1.06-2.30; *P = *0.024), respectively. The unadjusted HR for death-censored kidney graft loss in kidney recipients of donors aged 70 y and older compared with kidney recipients of donors aged 60–69 y was 1.42 (95% CI, 0.99-2.03; *P = *0.054; Table 5).

Multivariate Cox proportional hazards models were adjusted for recipient, donor, and transplant risk factors (Table 5). Recipient age, diabetes, donor age, and DGF were inversely associated with long-term death-censored kidney graft survival. The adjusted HRs and 95% CIs for death-censored kidney graft loss in kidney recipients of donors aged 70 y and older were 3.20 (95% CI 2.04, 5.00; *P < *0.001) and 1.75 (95% CI 1.18, 2.60; *P = *0.005) in recipients who received kidneys from donors aged 60–69 y, respectively, using kidney recipients of donors aged 45–54 y as a reference. The adjusted HR and 95% CI for death-censored kidney graft loss in kidney recipients of donors aged 70 y and older compared with recipients who received kidneys from donors aged 60–69 y were 1.89 (95% CI, 1.27-2.80; *P = *0.002).

## DISCUSSION

This study revealed that the outcome after transplantation of kidneys from DBD donors aged 70 y and older was acceptable, although kidney graft survival was inferior compared with that of kidneys from donors aged 60–69 and 45–54 y. However, these kidneys are generally transplanted into elderly recipients, where the alternative would have been a longer period on the waiting list or no transplantation at all. Therefore, we find our results in this cohort of elderly adults to be encouraging and advocate increased utilization of organs from elderly DBD donors.

Our findings are in accordance with previous reports that showed an increased risk of graft loss in kidneys from older deceased donors but with acceptable results.^10,12-16^ More recently, in 2021, Doucet et al^16^ compared the outcomes of transplant recipients aged between 18 and 69 y and those aged 70 y and older. They found that patients in the 18–69 y age group who received a deceased donor organ had graft survival rates of 93% at 1 y, 88% at 3 y, and 82% at 5 y, whereas patients aged 70 y and older who received a deceased donor organ had graft survival rates of 93% at 1 y, 80% at 3 y, and 74% at 5 y.^16^ In their study, there were 191 recipients aged 70 y and older, but contrary to our study, the mean DBD donor age to these recipients was only 50.0 ± 17.2 y, that is, very few received a DBD from a 70-y-old donor. Thus, our results are uplifting.

During the study period, the proportion of used DBD donors aged 70 y and older increased from 21% to 40% in the investigated donor groups (Figure 1). This trend has also been observed in other European transplant centers. In data from centers reporting to the Collaborative Transplant Study, the proportion of kidneys from 70-y-old donors more than doubled (6.7%–15.4%) from the period 1997–2006 compared with 2007–2016.^17^ A recently published study from the Scientific Registry of Transplant Recipients showed that waitlisted candidates aged 70 y and older during a 6 y observation period had 16% mortality and 39.4% chance of waitlist removal for those who became too sick to undergo transplantation or other reasons for waitlist removal beyond death and receipt of a transplant.^18^ The possibility of receiving a transplant was only 38.0%.^18^ By increasing the number of transplantations from elderly donors and not discharging these kidneys, the possibility of the elderly receiving a transplant increases.

In this study, a typical high-risk patient associated with worse kidney transplant outcomes was an older recipient with diabetes who had been on dialysis for a longer period before transplantation and experienced DGF after engraftment. Patients of increasing age who undergo kidney transplantation have higher mortality rates than younger recipients.^19^ The best way to address the possible survival benefit of kidney transplantation for older kidney recipients of DBD from donors aged 70 y and older compared with dialysis has been to compare survival rates between patients who were accepted for kidney transplantation and who had received a kidney transplant with those accepted for transplantation but who had not yet received a kidney transplant. Using these 2 comparative groups, studies have found that patient survival, regardless of age, is better with kidney transplantation than with dialysis.^5,6,20^

In our opinion, the most interesting aspect, as stated above, is the difference between elderly waitlisted patients undergoing transplantation and those who remain on the waiting list. Unfortunately, we do not have data to compare between these groups during the entire study period. However, our group addressed this question in 2010, focusing on the survival rate of waitlisted patients older than 70 y on dialysis between 1990 and 2005.^21^ We found an improvement during the study period, with a clear survival benefit for patients engrafted from 2000–2005 when compared with patients who remained on dialysis. No data indicate that this has changed. In Norway, there is still a high rate of acceptance of older recipients, even those with a substantial degree of comorbidity, for waitlisting. We also have a high rate of acceptance of DBD donors aged 70 y and older and rarely discard the offered kidney. The national policy is to transplant patients rather than stay on the waiting list to wait for a *better kidney*. Additionally, the waiting time for kidney transplantation in Norway is relatively short, although it has increased during the past decade. Maintaining a short waiting time is a way to optimize the “quality” of recipients. Fit recipients have an increased chance of postoperative success.

An increased donor age is associated with worse kidney graft survival.^8,9^ This is also a finding of the present study. Kuhn et al^15^ reported that kidney graft survival decreases with donor age, with an exponential risk of graft failure, especially in donors aged 70 y and older; however, this trend was already observed in donors aged 60 y and older. However, they reported an overall 5-y graft failure-free survival rate of 64.0% in recipients with donor grafts aged 70 y and older, which is consistent with our results. However, another recent study indicated that posttransplant results from elderly donors are improving, indicating that kidneys from donors aged 70 y in the current era perform equally well as kidneys from donors aged 60 y in the previous era, making it difficult to compare results from different periods.^17^

Most deaths attributable to cardiovascular disease occur in kidney recipients with diabetes.^22^ Diabetes, as a recipient comorbidity, is associated with inferior kidney transplant outcomes because of increased cardiovascular mortality.^22-24^ In this study, diabetes at the time of transplantation was associated with an increased risk of death and death-censored kidney graft loss. Furthermore, dialysis duration is associated with inferior kidney graft survival,^25-28^ which was also a finding of this study. By increasing the number of DBD transplantations, waiting times are reduced and the overall results will improve. This would be a win-win situation.

Prolonged CIT is associated with impaired survival of the kidney grafts.^29^ CIT was not associated with death-censored kidney graft loss in the present study, probably because of the relatively short CIT and the lack of group differences. This result is consistent with the findings of Echterdiek et al.^30^ They showed that CIT, if kept ≤18 h, has no significant impact on kidney transplant outcomes, even in recipients of kidneys from deceased donors aged 70 y and older.

It is known that kidney transplant recipients experiencing DGF have impaired short- and long-term kidney transplant outcomes.^29^ The results of the present study confirm this finding. The fact that we have 1 transplant center cowering for the entire nation and that the transplant surgeons are responsible for “procuring” the organs from all 28 donor hospitals leads to very good logistics, that is, a short CIT. A short CIT is important when using organs for old DBDs; hence, when planning logistics for transplantations, extra resources are often activated to keep the CIT as short as possible. This may partially explain the low DGF rates observed in this study.

The strength of our study is that it was a single-center study addressing the results from recipients who received transplants during a period of consistent immunosuppressive therapy, with complete and long-term follow-up. No patient was lost to follow-up, which is also a strength. A limitation of this study was the relatively small number of included patients. Therefore, the analysis may have been underpowered to detect smaller differences between groups. Although we adjusted for possible confounding factors, uncontrolled factors such as patient comorbid conditions may still confound our results. Furthermore, factors such as donor renal function, donor BMI, CIT, and DGF were favorable in this study, and these factors may mean that our results may not be easily translated to a system with greater geographic challenges and a less healthy overall population. Additionally, the current study did not consider healthcare costs, which is an important factor when evaluating the expanded use of higher-risk donors. Therefore, future studies should address this research gap. Furthermore, this study was conducted at a single transplant center in Norway. The results may reflect specific practices at our center and may not be applicable to other populations and healthcare systems, and the results may not extend to populations with greater diversity, varying comorbidities, or different healthcare access levels.

In conclusion, kidney recipients of DBD deceased donors aged 70 y and older experienced inferior kidney graft survival compared with kidney recipients of deceased donors aged 60–69 and 45–54 y, yet kidney transplantation with donors aged 70 y and older can succeed and should be an option for a selected group of older recipients.

**TABLE 5.** HRs for death-censored kidney graft loss by donor age group

Variable	Unadjusted			Adjusted		
	HR	95% CI	*P*	HR	95% CI	*P*
Recipient age (per year)	0.99	0.98-1.01	0.28	0.98	0.96-0.99	0.001
Male recipient	1.19	0.85-1.65	0.31			
Diabetes	1.46	1.06-2.01	0.021	1.42	1.03-1.95	0.034
CMV seropositive recipient	0.80	0.57-1.12	0.19			
Time on dialysis (per year)	1.10	1.02-1.20	0.019	1.07	0.98-1.17	0.11
Donor age group						
45–54 y		Reference			Reference	
60–69 y	1.56	1.06-2.30	0.024	1.75	1.18-2.60	0.005
≥70 y	2.25	1.51-3.36	<0.001	3.20	2.04-5.00	<0.001
Male donor	0.95	0.70-1.30	0.77			
≥1 HLA-DR mismatches	1.17	0.84-1.62	0.36			
HLA class I and/or II PRA	1.39	0.97-2.01	0.074	1.38	0.95-1.99	0.088
Cold ischemia time (per hour)	0.99	0.96-1.02	0.62			
Delayed graft function	2.14	1.45-3.17	<0.001	1.95	1.31-2.90	0.001

CI, confidence interval; CMV, cytomegalovirus; HR, hazard ratio; PRA, panel-reactive antibody.
